# Non-colorectal liver metastases: A review of interventional and surgical treatment modalities

**DOI:** 10.3389/fsurg.2022.945755

**Published:** 2022-11-02

**Authors:** Daniela Kniepeiss, Emina Talakić, Rupert Horst Portugaller, Michael Fuchsjäger, Peter Schemmer

**Affiliations:** ^1^ General, Visceral and Transplant Surgery, Medical University of Graz, Graz, Austria; ^2^ University Transplant Center Graz, Medical University of Graz, Graz, Austria; ^3^ Department of Radiology, Division of General Radiology, Medical University of Graz, Graz, Austria; ^4^ Division of Neuroradiology, Vascular and Interventional Radiology, Department of Radiology, Medical University of Graz, Graz, Austria

**Keywords:** liver metastases, surgical oncology, interventional radiology, outcome, patient selection, highly specialized centers

## Abstract

Liver metastases (LM) occur in up to 90% either simultaneously with the diagnosis of the primary tumor or at a later time-point. While resection of colorectal LM and resection or transplantation of neuroendocrine LM is part of a standard therapy with a 5-year patient survival of up to 80%, resection of non-colorectal and non-neuroendocrine LM is still discussed controversially. The reason for it is the significantly lower survival benefit of all different tumor entities depending on the biological aggressiveness of the tumor. Randomized controlled trials are lacking. However, reviews of case series with ≥100 liver resections are available. They show a 5-year patient survival of up to 42% compared to only <5% in patients without treatment. Risk factors for poor survival include the type of primary tumor, a short interval between resection of the primary tumor and liver resection, extrahepatic manifestation of the tumor, number and size of the LM, and extent of liver resection. Overall, it has recently been shown that a good patient selection, the technical advances in surgical therapy and the use of a risk score to predict the prognosis lead to a significantly better outcome so that it is no longer justified not to offer liver resection to patients with non-colorectal, non- endocrine LM. Since modern therapy of LM is multimodal, the optimal therapeutic approach is decided individually by a multidisciplinary team consisting of visceral surgeons, oncologists, interventional radiologists and radiologists as part of a tumor board.

## 1. Introduction

Liver metastases (LM) are common in different types of malignant diseases, either at diagnosis of the primary tumor or at a later time point. Although resections of colorectal liver metastases and resections or transplantation for neuroendocrine liver metastases are considered standard therapy (1–3) and are associated with significant improvement in 5-year patient survival of up to 80%, resections of non-colorectal and non-neuroendocrine LM remain controversial because of the difference in underlying tumors. This is because the survival benefit may be significantly lower when all possible tumor entities are considered, depending on the biological aggressiveness of the tumor. Although there are no randomized controlled studies to support the decision of liver surgery as a treatment option, there are case series with ≥100 liver resections of LM from non-colorectal cancers and non-neuroendocrine tumors (4, 5). They documented a 5-year survival rate of up to 42%, with a median survival of up to 49 months. The 5-year recurrence-free survival rate was up to 29%, with a median recurrence-free survival of up to 21 months. Without treatment, the 5-year survival rate is <5% only (6). Risk factors for poor survival included type of the primary tumor, a short time interval between primary tumor and liver resection, extrahepatic tumor manifestation, LM number and size, and extent of liver resection.

Recent data have shown that good patient selection, technical advances in surgical therapy, and the use of a risk score to assess prognosis have resulted in a significantly better outcome (7–10). Therefore, it is no longer justifiable to withhold the option of liver resection from patients with non-colorectal, non-endocrine metastases in the future. Multidisciplinary and multimodal approaches achieved the best results, whereas treatment approaches without surgery could only prolong survival by a few months (10).

Liver transplantation (LT) is an established procedure for the treatment of LM from neuroendocrine neoplasia, provided that the established criteria (11) are met. In recent years, a survival benefit has also been demonstrated for colorectal LM (12). Other indications occur only in single case reports and have no evidence (13, 14).

The therapeutic significance of locoregional treatment methods is limited. When used alone, such approaches have more of a palliative significance. The indication for local treatment is indivizualized for each patient based on biology, localization, and disease burden of the LM (15).

Because modern therapy for liver metastases is often multimodal, the optimal treatment approach is determined individually by a multidisciplinary team of visceral surgeons, oncologists, interventional radiologists, and radiation therapists in a tumor board setting.

This article presents an overview of interventional and surgical treatment methods for non-colorectal LMs. The topic is highly relevant in multidisciplinary tumor boards, but it is challenging to be clearly presented in a review. Therefore, we focussed on state-of-the-art therapy options on the one hand and on the other hand, current data with their oncological outcomes were added. With the information of this review, it should be easier to make treatment decisions for patients with non-colorectal LMs in a multidisciplinary tumor board.

## 2. Interventional therapy

In clinical use, there are a number of modalities of locoregional therapy based on different physical principles. They can be performed percutaneously, laparoscopically, or in the context of open surgery. Minimally invasive percutaneous procedures are performed using imaging modalities such as ultrasound (US), computed tomography (CT), or magnetic resonance imaging (MRI) for guidance. Ablation techniques are more difficult to perform when the visibility of a tumor in imaging is limited (16). The feasibility of local ablation techniques is determined by factors such as tumor size, number, and location. Such approaches may be indicated in rare cases when resection and/or transplantation are contraindicated; they are also part of multimodal therapeutic approaches (17).

### 2.1. Relevance of interventional therapy

Cross-sectional imaging techniques (CT, MRI) allow three-dimensional treatment planning, which is particularly relevant for overlapping ablation zones in larger or anatomically complex tumor entities. Stereotactic navigation systems and targeting devices can greatly improve the results of ablation procedures. Ablation is a safe and practical alternative to surgery that not only spares the parenchyma but is also associated with fewer side effects and lower post-interventional costs. However, such interventional techniques are limited by the different modalities of evaluation for tumors that are difficult to detect or are associated with discordant tumor stages. The relevance of endovascular techniques for palliative treatment of liver metastases has only been discussed in case reports, abstracts, and retrospective cohort studies, and long-term results are not yet available. Nevertheless, early results of SIRT for the treatment of melanoma are promising, when showing prolonged survival with low toxicity in small patient cohorts (16).

### 2.2. Local ablation procedures

In minimally invasive procedures, the tissue is specifically damaged by heating (radiofrequency ablation, microwave ablation, laser-induced thermotherapy), freezing (cryotherapy), irradiation (percutaneous stereotactic irradiation, interstitial brachytherapy) or electrical charges applied to cell membranes (irreversible electroporation, IRE). To be eligible for ablation, lesions should ideally be smaller than 3 cm and be sufficiently distant from vulnerable structures (gallbladder, bile duct, diaphragm, pericardium). More than one lesion can be ablated at a time. Due to the limited supply to the liver parenchyma and the time required, no more than three non-colorectal or five colorectal metastases are treated. Given the risk of local recurrence after radiofrequency ablation, other regional therapies are usually preferable for tumors larger than 3 cm. Previous chemoembolizations may help shrink larger tumors, making thermoablation feasible. To avoid residual tumor tissue (18), multiple ablation probes must be placed simultaneousy or sequentially overlapping when thermoablating large tumors.

### 2.3. Endovascular techniques

In endovascular treatments of highly vascularized tumors, embolizing and therapeutic agents can be delivered into the target vessels, causing ischemia in the tumor and enhancing the local effect of administred chemotherapeutic agents. Locoregional therapy for liver metastases is not effective when there is untreatable metastatic growth outside the liver (18). An overview of principles, advantages and disadvantages of locoregional treatments is displayed in Table 1.

**Table 1:** Overview of locoregional treatments, their principles, advantages and disadvantages.

Locoregional treatments				
Method	Technique	Principle	Advantage	Disadvantage
RFA	High-frequency alternating current (450–500 kHz)	Frictional heat (50–105°C)	Necrosis and cell death to surrounding tumor cells	“heat sink effect”
MWA	Elektromagnetic fields (900 MHz–2,4 Ghz)	Cell destruction based on oscillation of water molecules	No “heat sink effect”	Similar outcome as RFA
Cryoablation	Cryo-probe	Cryo-probe with liquid nitrogen (−196°C) is placed in the lesion	For ablating tumors that are located close to vital structures	Risk of bleeding and cryoshock
IRE	Electrical fields to cause the permanent permeabilization of cell membranes	Electrodes with short high-frequency pulses to induce cell death	No “heat sink effect” For tumors located close to vascular structures or bile ducts	General anesthesia, Muscle blockade
TAE	Particles 40–700 µm	Highly selective catheterization of segmental or subsegmental arteries	Absence of side-effects compared to TACE	Lower efficacy compared to DEB-TACE
TACE	Chemotherapeutic agents into the tumor	Targeted intraarterial administration	Reduced side effects compared to systemic administration	Side effects of cell death
DEB-TACE	Drug-eluting bead	Release of chemotherapy within the tumor	Superior to TACE in terms of efficacy, system toxicity and tolerability	Patients with impaired liver function
SIRT	Intraarterial microsheres (30 µm), Y-90 beta emitter	Y-90 beta emitter causes the death of tumor cells through brachytherapy	Radiotoxicity	Minimal invasiv

RFA, radiofrequency ablation; MWA, microwave ablation; IRE, irreversible electroporation; TAE, transarterial embolization; TACE, transarterial chemoembolization; DEB TACE, drug-eluting bead TACE; SIRT, selective internal radiation therapy.

### 2.4. Oncologic outcomes reported with locoregional treatments

Surgical resection is the best oncologic treatment option for patients with defined tumors but is limited by the risk of post-surgical liver failure. In patients with low future liver remnants (FLR), compensatory hypertrophy of the contralateral liver lobe can be achieved by portal vein embolization prior to surgery to allow a surgical resection (19).

When surgical resection is not feasible, further therapeutic options comprise different methods of locoregional therapies. In the absence of clear treatment algorithms, the use of individual interventional therapeutic procedures often depends on the specific clinic or patient situation. In recent years, a greater research focus has been placed on local treatments for non-colorectal cancers, and recent studies have confirmed promising results following local treatment of non-colorectal liver metastases. In the following, therapeutic approaches with promising results are described.

#### 2.4.1. Radiofrequency ablation (RFA)

There are only few studies on RFA in non-colorectal LM. Hwang et al. (20) evaluated the efficacy of RFA in patients with metachronous LM of gastric cancer. Systemic chemotherapy was combined in most of the patients. The results of this study showed, that the success of RFA was limited to patients with a single, unilobular metastases and combined chemotherapy was decisive for overall survival. The effect of stereotactic RFA in patients with breast cancer LM was evaluated in a recent study (21). A benefit concerning the overall survival compared to no treatment was described, but there was no significant survival benefit in comparison to liver resection. In case of surgical not treatable LM or patient comorbidities, RFA might be an alternative therapeutic option. Similar results were found in an analysis of 22 patients with LM from uveal melanoma, which were treated with RFA and compared to patients with liver resection (22). The number of LM was lower in the RFA group, but the median overall survival and disease-free survival between both groups showed no significant difference. Hence, RFA can be used to treat LM from uveal melanoma in order to save parenchyma of the liver.

#### 2.4.2. Microwave ablation (MWA)

A prospective randomized trial comparing MWA and RFA for the treatment of LM was performed recently (23). Twenty-six patients were treated with MWA and 24 patients with RFA. LM were of different origins: colon cancer, breast cancer, pancreatic adenocarcinoma, ovarian carcinoma, neuroendocrine neoplasia, esophageal cancer and uveal melanoma. In all interventions with RFA and MWA technical success was achieved. The one and two-year survival rates of both groups showed no significant difference and there was no difference in relation to the index tumor. Both treatments were safe and there were no differences for major complications between both groups. Hence, MWA could be an alternative therapy for patients with LM from different cancers.

#### 2.4.3. Transarterial chemoembolization (TACE)

TACE is a safe method for the treatment of LM. The effect of TACE is described controvers in the literature and therefore TACE is suggested as an option in those patients who are not candidates for curative treatments such as resection. Future efforts are aimed at being able to treat larger LM successfully. A recent study evaluated TACE combined with percutaneous thermal ablation in LM larger than 3 cm (24) and achieved high local control rate with a local tumor progression rate at 12 months with 13%. In addition, an improvement in the results should be sought through the further development of the technology itself (25, 26).

#### 2.4.4. Selective internal radiation therapy (SIRT)

SIRT has been used to treat primary hepatic tumors or LM from colorectal and neuroendocrine tumors. There are only few data concerning non-colorectal and non-neuroendocrine LM. In unresectable chemoresistant LM of breast cancer, SIRT achieved a median overall survival up to 13.6 months (27). The effect of SIRT in breast cancer in comparison to TACE, RFA and hepatic resection is displayed in Table 2. Results of SIRT for LM from other cancers are less encouraging. Additionally, clinical guidelines are lacking.

**Table 2:** The role of RFA, SIRT, TACE and hepatic resection (HE) in patients with breast cancer.

LM of breast cancer					
Technique	Advantage/Disadvantage	Patients (*n*)	Size (cm)	OS (months)	DFS (months)
RFA	Low morbidity, repeatability, insufficient local control in the case of large tumors	203	2–2.5 (0.5–5)	11–60	8–24
SIRT	Low morbidity, palliative	380	NR	4–14	3.2/NR
TACE	Low morbidity, palliative	71	2.8 (1–8)	10–28	3.3/NR
HR	Good local control, high morbidity	1173	1.8–5.1 (0.4–19)	24–116	11–53

OS, overall survival; DFS, disease-free survival.

In conclusion, locoregional treatments could offer an alternative therapy for non-colorectal LM. Some studies have already shown comparable survival data to that of liver resection, and therefore these treatments could represent an alternative especially for unresectable LM or patients with comorbidities. But, their finally role in metastatic liver disease and the optimal patient selection has to be defined. Therefore, therapeutic algorithms should be developed and evaluated in prospective trials.

## 3. Surgical therapy

### 3.1. Liver resection

Only a small and very heterogeneous group of patients with non-colorectal LM is eliglible for resection. Most of of these patients have multiple LM or even metastatic liver with extrahepatic tumor manifestation at diagnosis, excluding resection as a successful treatment option. Apart from the obvious contraindications to liver resection, the selection criteria defined by Adam et al. (6) still apply. Their prognostic significance has been clearly presented and validated in a recent multicenter study (28). The scoring system takes into account criteria such as type of primary tumor, extent of liver resection, radicality, or recurrence-free interval. The resulting 5-year survival provides helpful information for decision-making, which should preferably be done in a specialized, interdisciplinary tumor board. In the individual risk-benefit assessment, the probability of complete local resection (R0) must also be estimated.

Due to the great heterogeneity of LM from different tumors and the associated range of treatment concepts and prognoses, the section below is only divided according to localization of the primary tumor and does not take any other criteria into account.

#### 3.1.1. Gastrointestinal cancer

##### 3.1.1.1. Stomach cancer

Although stomach cancer is the second most common malignancy worldwide, LM resection is only performed in a small number of patients. In most cases, when metastases appear, the disease is already too advanced with concomitant extrahepatic metastases, peritoneal carcinomatosis, or multiple LN metastases. The largest studies with more than 40 patients have shown 3-year and 5-year survival rates of up to 51% and 42%, respectively (29). Patients with the following risk factors have a poor prognosis:

• Multiple LMs
• Synchronous LMs
• Large LM diameter
• Serosa invasion by primary tumor
• Positive LN status (*N* > 2) of primary tumor

##### 3.1.1.2. Gastrointestinal stromal tumors (GIST)

The introduction of tyrosine kinase inhibitor therapy (TKI) has significantly improved the treatment options and prognosis for GIST. The 3-year and 5-year survival rates of up to 90% and up to 76%, respectively (30), have been reported after resection of GIST-related LM. Patients with the following risk factors have a poor prognosis:

• Positive resection margin (R1)
• Non-surgical therapy
• Extrahepatic manifestation
• No use of TKI
• Progression under TKI
• Males

Surgical treatment remains controversial, although recent data have shown that a combination of TKI and surgery is associated with a better prognosis than TKI monotherapy (31).

##### 3.1.1.3. Other primary gastrointestinal tumors

Esophageal cancer and pancreatic adenocarcinoma are associated with LM, which are reasonable to resect, especially since they typically manifest at the same time and in greater numbers. Prognosis is very poor, with a median overall survival of up to 20 months (32); a survival benefit after resection has been demonstrated only in rare cases (33). The simultaneous resection of no more than 3 synchronous LMs along with the primary tumor of a ductal pancreatic adenocarcinoma can achieve a long-term survival rate of 10%, which is a significante advantage over palliative therapeutic approaches (34).

Specific tumor types of the panceas, such as NET (35), acinar cell carcinoma (36), or Frantz tumors (37), as well as tumors of the duodenum (38) and small intestine (6), have a significantly better prognosis if they metastasize into the liver. LMs of these tumors should be treated with multimodalitytherapy concepts based on the recommendations of an interdisciplinary tumor board.

#### 3.1.2. Breast cancer

Breast cancer is the most common malignant tumor in women and leads to distant metastases in more than 50% of cases. Isolated LMs are rather rare. The 3-year and 5-year survival rates after resection of breast cancer LMs reported in the literature range from 49%–68% and 27%–53%, respectively. In high-volume centers, postoperative mortality has been reported to be less than 5% and even dropped to 0% in a series of 41 patients (39).

Unfavorable prognostic factors include:

• Short interval between primary tumor and onset of LMs
• Negative expression of hormone receptors
• Poor response to chemotherapy prior to surgery
• Multiple LMs
• Positive resection margin
• No trastuzumab therapy (HER-2-negative patients)

Patients without unfavorable prognostic factors may have a significant survival advantage after liver resection, especially for metachronous, solitary, resectable LM. In individual cases, multimodal approaches can also achieve good results in patients with extrahepatic metastases, who should therefore not be considered absolute contraindications.

#### 3.1.3. Malignant melanoma

In the 4 largest studies with more than 40 patients, very poor outcomes were observed after liver resection of LMs for malignant melanoma, with a 5-year survival rate of almost 20% and a median survival time of up to 28 months (40).

Unfavorable prognostic factors:

• Short interval between primary tumor diagnosis and occurrence of LMs
• Positive resection margins (R1) of the primary tumor
• *N* > 4 LMs
• Cutaneous melanoma
• No chemotherapy before surgery

Cutaneous melanoma metastasizes to the liver in only 10%–20% of cases, but usually produces simultaneous distant metastases in other organs. Uveal melanomas metastasize to the liver in up to 80% of cases, but usually has no additional distant metastases. Liver resection should be performed as part of a multimodal approach in combination with chemotherapy. Recurrences have been reported to occur within the first year after liver resection. Cutaneous melanoma tend to recur outside of the liver, whereas uveal melanomas had a high rate of intrahepatic metastases. However, due to the poor response to chemotherapy and frequent simultaneous extrahepatic metastases, prognosis tends to be poor.

#### 3.1.4. Sarcoma

5-year survival rates of up to 46% have been observed after resection of LMs from sarcoma. Risk factors for poor prognosis include a time interval of less than 24 months between the primary tumor and LMs, non-GIST leiomyosarcoma, extrahepatic manifestation, and positive resection margins in the primary tumor (41). Because the risk of intrahepatic recurrence is significantly higher after radiofrequency ablation than after resection (85% vs. 50%), the latter should be preferred in patients without negative risk factors. A multimodal therapy approach is not recommended due to poor response to chemotherapy.

#### 3.1.5. Cancers of the urogenital tract

LMs may occur in rare cases in association with renal cell carcinoma, ovarian cancer, and testicular cancer. After resection of LMs for renal cell carcinoma, 5-year survival rates of up to 62% have been reported. Unfavorable prognostic factors include:

• Positive resection margin (R1)
• Positive LN of the primary tumor
• Synchronous LMs
• Short disease-free interval
• Extrahepatic manifestation

Compared to conservative approaches, LMs resection can lead to good therapeutic outcomes (42). The 5-year survival rate after resection of LMs from cancers of the genital tract is up to 51%, slightly lower than that of renal cell carcinoma with the same constellation (6). In testicular cancer patients, adjuvant chemotherapy before liver resection improves the 5-year survival rate from 26% to 48% (43).

#### 3.1.6. Neuroendocrine neoplasia

Neuroendocrine neoplasia occurs rarely and are highly heterogeneous in terms of localization, malignancy, and prognosis. At the time of diagnosis, liver metastases are already present in up to 95% of cases. The risk of developing LMs depends on the degree of differentiation and proliferation, but also on the localization of the primary tumor. Esophageal neuroendocrine neoplasia has the highest risk at 49.4%, while neuroendocrine neoplasia of the appendix has the lowest risk at 2.8% (44). The LMs of neuroendocrine neoplasia can occur in different manifestations, which affect the corresponding treatment options and outcome. In approximately 20%–25% of cases, metastases occur in only one liver lobe. In 10%–15% of cases, one liver lobe is primarily affected while other lobes show satellite lesions, and in 60%–70% of cases, metastases are diffusely distributed throughout the liver (45). Depending on the extent of the LMs, treatment options for patients with negative findings in the surrounding tissue include liver resection or LT.

Resection with curative intent is the gold standard for LMs of neuroendocrine neoplasia, with a 5-year survival rate of 60%–80%, low mortality (0%–5%) and morbidity (30%) (46). Patients whose LMs were not resected have a very poor prognosis with a 5-year survival rate of only 30% (47). The conditions for resection with curative intent are limited and are present in only 10%–25% of patients (46).

Selection criteria for liver resection:

• WHO- neuroendocrine neoplasia G1–2
• Primary tumor removal (if known)
• Solitary metastases or metastatic growth restricted to a single liver lobe
• Absence of extra-abdominal metastases or peritoneal carcinomatosis

It is not recommended to resect LMs from a WHO- neuroendocrine neoplasia G3, but the procedure may be considered individually as part of a multimodality therapeutic concept with palliative intent.

### 3.2. Liver transplantation

LT is currently contraindicated for non-colorectal LM except in patients with neuroendocrine neoplasia, subject to defined selection criteria. The first LT outcomes for neuroendocrine neoplasia were disappointing, resulting in a 5-year survival of 36%–67% due to inadequate patient selection (48). By establishing selection criteria, LT became the standard therapy with significantly improved survival (5-year survival rate of 97.2%). The selection criteria (11) published by Mazzaferro in 2007 for LT in patients with neuroendocrine neoplasia LMs still serve as the basis of recommendations.

Favorable prognostic factors for LT:

• WHO neuroendocrine neoplasia G1–2
• Primary tumor drains into the portal vein system and was removed for treatment
• Liver tumor load <50%
• Stable disease
• Age <55

Unfavorable prognostic factors for LT:

• WHO neuroendocrine neoplasia G3
• Primary tumors not draining into the portal vein system
• Primary tumors that are not GEP- neuroendocrine neoplasia
• Other medical or surgical contraindications for LT

Only a very small proportion of neuroendocrine neoplasia patients (about 1%) meet these criteria. When the above Mazzaferro criteria are met, a 5-year and 10-year survival rate of 97.2% and 86.9%, respectively, can be expected (3, 49). LT is therefore a curative treatment method for patients who meet the selection criteria. Patients with neuroendocrine neoplasia -related LMs must be referred to a highly specialized transplant center and evaluated for a possible LT.

## 4. Conclusion

LMs of colorectal cancers usually originate from the portal vein circulation or intestinal lymph node tracts. Compared with non-colorectal LMs that spread *via* the systemic circulation, they are therefore associated with lesser tumor spread. This may be a possible reason why the resection of colorectal LMs has a clear advantage for patient survival, whereas outcomes are significantly worse in patients with non-colorectal LMs (5-year survival rate 80% vs. 42%). Cancers that metastasize to the liver *via* the systemic circulation also frequently have a multilocal metastatic growth pattern with a correspondingly poor prognosis.

Because of their heterogeneity, there is still no standardized treatment approach for non-colorectal LMs, and no randomized controlled trials are available. Non-surgical approaches do not result in satisfactory therapeutic outcomes and are usually associated with a gain in survival of only a few months.

In recent years, advances in tumor biology and the development of multimodal therapy concepts with personalized chemotherapy regimens have led to improvements in cancer therapy. Improved equipment technology has further enhanced available interventional radiology procedures and contributed to local control of LMs. Advances in surgical technique and perioperative care have reduced patient morbidity and mortality after liver resection to approximately 30% and <5%, respectively (50–52), and have enabled a 5-year survival rate of >80% after LT, justifying surgical treatment of non-colorectal LMs.

In order to predict survival after hepatic resection in patients with non-colorectal LM, Wakabayashi et al. developed a predictive model in the context of a multicenter analysis (51). Patients with LM from different primary tumor sites were analyzed with a primary endpoint of 5-year survival. R0 resection was performed in 85% and the overall survival at 5 years was 41%. A predictive model was evolved with patients with low, intermediate and high risk related to tumor pathology, timing and kind of metastases and curative resection. The expected 5-year survival rates depended on the risk score (63% low risk, 38% intermediate risk, 21% low risk). In case of low risk, results comparable to LM of colorectal cancer could be achieved.

Embedded in partially personalized and multimodal concepts, surgical therapy is considered the standard procedure for non-colorectal LMs with appropriate patient selection, which has yet to be defined and confirmed in randomized, controlled trials. However, there are some locoregional treatments which have shown similar results as liver surgery (53–55) and therefore should be added as therapeutic options. As expression of personalized medicine less invasive local treatment methods should be considered if appropriate.
